# In vitro evaluation of dental color stability using various aesthetic restorative materials after immersion in different drinks

**DOI:** 10.1186/s12903-023-02719-3

**Published:** 2023-01-29

**Authors:** Tavga Mustafa Faris, Rukhosh Hasan Abdulrahim, Mohammed Abdalla Mahmood, Gollshang Ahmad Mhammed Dalloo, Sarhang Sarwat Gul

**Affiliations:** 1 Department of Conservative Dentistry, College of Dentistry, University of Sulaimani, Sulaimaniyah, 0046 Iraq; 2 Department of Basic Science, College of Dentistry, University of Sulaimani, Sulaimaniyah, 0046 Iraq; 3 Department of Periodontology, College of Dentistry, University of Sulaimani, Sulaimaniyah, 0046 Iraq; 4 Presidency, Sulaimani Polytechnic University, Sulaimaniyah, Iraq

**Keywords:** Color stability, Esthetic restoration, Nano-hybrid, Glass ionomer, Micro-hybrid

## Abstract

**Background:**

Currently, the demands for restorations have increased considerably; thus, improvements and modifications have been made in dental composite technologies especially using materials that have been claimed to improve color stability.

**Objective:**

This study aimed to determine the effect of various solutions on the color stability of different restorative materials in vitro study.

**Methods:**

This study used three types of esthetic restorative materials. The samples comprised 45 discs, including 15 micro-hybrids, 15 nano-hybrids, and 15 glass ionomers). These discs were immersed in various beverages an hour a day for one month at room temperature. The color stability was measured using a spectrophotometer before/after immersion (days 7 and 30).

**Results:**

We realized a significant difference in color change with Coca-Cola and tea-milk solution after seven days and 30 days (*p* < 0.05). However, no significant difference was found in the samples immersed in DM after seven days and 30 days (*p* > 0.05). The highest value of lightness (∆L) and stainability (∆a) was seen in micro-hybrid after 30 days of immersion in tea-milk solution (− 12.16 ± 1.74 and 11.4 ± 3.82, respectively), while most samples had a positive ∆b value.

**Conclusion:**

After one month, the tea-milk solution affected the color stability of all used restorative materials. In addition, micro-hybrid had higher color stability than nano-hybrid and glass ionomer.

## Introduction

Generally, there is a significant association between the characteristics of the most negative experiences of dental treatment and increased dental issues, including physical discomfort, fear, anxiety, and pain, especially in children, teenagers, and young adults, which instigates the patients to expect similar suffering during consequent visiting [1].

Today, people complain of common dental issues such as dental caries, cavities, and root canal infections. All these problems are due to bacterial infection of the teeth, which damages the tooth structure [1]. To treat the affected tooth, dentists recommend the removal of dental caries and filling the cavities with suitable materials. In addition, dental amalgams percolate from the tooth during chewing and affect human health. Compared to previous filling materials, polymer composites are widely used as dental restoration materials due to their superior properties, such as biocompatibility, excellent esthetics, antibacterial and non-toxic properties. In addition, it demonstrates good physical, mechanical, thermal, and tribological properties [2].

Aesthetic restorative materials thoroughly mimic the dental structure as they enhance and maintain natural tooth whitening, transparency, texture, and surface staining stability for an extended period [3]. The initial restorative material first presented to dental work in the early seventies was glass ionomer, widely spread worldwide due to its promising adhesive and fluoride-releasing characteristics [4].

Subsequently, composite resin, hybrid materials, porcelain veers, compomers, and ceramic crowns were developed to overwhelm the disadvantages of glass ionomer and maintain their clinical benefits. Still, they also had some potential drawbacks, including polymerization shrinkage, secondary caries, plaque formation, limited releasing time, and color instability [2, 5].

Additionally, many other types of tooth restoration materials are commonly used; however, their quality, physical, and mechanical features should be high enough not to be easily subjected to color change after a short period, although most have a similar appearance to the tooth substrate. Generally, their disadvantages are color alteration, fracture, poor wearing, leakage, and polymerization shrinkage [6–8].

Moreover, it has been determined that the surface of aesthetic restorative materials is affected by drinks (coffee, tea, and wine), smoking, physiochemical stress, and some foods, which are mainly caused by either absorption or adsorption of coloring substances available in the diet and beverages [9]. On the other hand, superficial degradation on the composite surface produced by diets and drinks reduces its hardness and roughness, which leads to external coloring [10, 11].

Half of all dental restorations fail within ten years [12]; thus, the physical assets and quality of aesthetic restorative materials that are widely used in the dental field can be improved through manipulation of their matrix, size, strength, type, and quantity of filler particles, especially nanocomposite that is used for anterior and posterior teeth restoration [13–15].

Biomaterials have been widely used in dental composite materials or implants and their physical, mechanical, wear, and biocompatibility characteristics must be evaluated for clinical application. Therefore, extensive research has been committed to establishing various scientific methods for customizing the microstructure of dental biomaterials [1].

Moreover, various rechargeable dental nanocomposites with sufficient remineralization ability but with persistent ion release limitation [16] have also been intended and examined by different research groups in other countries for dental restoration with higher dental care than micro-hybrid composites such as nano-hybrid (nanofillers) that have advanced optical and physical features [17–21].

These tooth-colored resin-based dental composite materials have been introduced continuously to restore the esthetic and function of human teeth and meet the demands of the ever-increasing human population with increased longevity [22].

Therefore, this study aimed to measure the color stability of micro-hybrid, nano-hybrid, and glass ionomer restorative materials after immersing in tea-milk solution, Coca-Cola, and distilled water (DW), an hour a day for 30 days. The null hypothesis includes no differences in color stability of tested materials following immersing in the selected solution.

## Materials and methods

### Samples and study design

We prepared 45 disc-shaped samples (10 mm diameter × 3 mm thickness) from different esthetic materials. A package of the tablet has been used with dimensions that match our sample size, including 15 samples from micro-hybrid (Rio Javama-120-45007-Toledo Espana, Spain), 15 samples from nano-hybrid (Itena reflects-75116, France), and 15 samples from glass ionomer; shade A2 (Riva Self Cure, Australia). The samples were invented into a customized plastic container after condensation of their materials. The nanohybrid composite was made up of a micro-sized diameter of (0.3–1 µm) and nanosized diameter (0.02–0.05 µm) fillers while micro-hybrid particle size (0.7–2 µm) fillers. First, micro-hybrid and nano-hybrid samples were light-cured (Light Cure, COXA, DB686, China) for 20 s based on the instruction protocol. Then, all samples were polished with super-snap polishing discs (Nova Twist, Germany) and preserved in DW overnight at 25 °C.

### Beverage preparations

We used tea-milk solution (Saadeldeen, Srilanka) and Coca-Cola (Kirkuk, Iraq) as beverages and DW as a control group. Regarding the tea milk preparation, about 4.0 g of dry powdered black tea was added to 0.3 L of boiled water in a small beaker. Then, 0.1 L milk solution and 10 g fine-white sugar were added and left to boil for 5 min. Whereas Coca-Cola was used as a carbonated drink directly without any preparation, the bottle lid was thoroughly tightened to prevent carbonic gas escape. The DW was directly collected from the distillation, while a freshly prepared tea-milk solution with immediately opened Coca-Cola bottles was used daily for the test.

### Specimens immersion

Samples of each group (n = 15) were subdivided into three subgroups (n = 5) and were immersed separately in either DW (control), tea-milk solution (treatment I), or Coca-Cola (treatment II) for an hour each day at 37 °C, then took them out with forceps, wiped properly until dryness with clean tissue paper and stored in a sterile place at room temperature for consecutive days. The duration of the study was exactly 30 days.

### Color alteration estimation

The specimen's color was tested before exposure (baseline; T0), after 7 days’ exposure (T7), and after 30 days of exposure (T30) using a calibrated reflectance spectrophotometer Vita Easy Shade V (Vita, Zahnfabrik, Germany), the new fifth generation of Easy Shade, was used in this study to measure the color changes. This is a more innovative and precise device that, due to the LED technology, is unaffected by ambient conditions and produces objective and reliable measurements. The probe tip illumination diameter is 5 mm. Software version v507k (serial number: H55328, light source: white LED D65) was used.

Briefly, each sample was put on the assessing head of the device and enclosed with the dark lid, then the device was turned on, and the mean color quantity of the sample (ΔL*, Δa*, and Δb*) was automatically obtained. Later on, the color difference (ΔE) was calculated for each specimen. Finally, CIELAB (ΔEab) was used for measuring color differences as follows and analyzed:

$$\Delta {\text{E}}\left( {{\text{L}}*{\text{a}}*{\text{b}}*} \right) = \left[ {\left( {\Delta {\text{L}}*} \right)^{{2}} + \left( {\Delta {\text{a}}*} \right)^{{2}} + \left( {\Delta {\text{b}}*} \right)^{{2}} } \right]^{{{1}/{2}}}$$

where L* denotes the lightness of the material, a* indicates a change in hues across the red-green axis, and b* shows the difference in hues across the yellow-blue axis. Triplicate measurements were done at different sites of each sample to get a valuable average.

### Statistical analysis

All material data will be analyzed using a statistical software package (SPSS, Inc., Chicago, USA, version 26). The distribution of the samples according to the type of drink and time of immersion was arranged on tables. The mean value of each material's prevalence of color change was calculated, and then their means were compared using the Chi-square test. The *P* value of < 0.05 was considered significant. ANOVA test is used to show the difference in the material behaviour when exposed to different solutions.

## Results

In the present study and after processing the samples in different solutions, the color change was measured on the first day (baseline), the seventh day, and then after 30 days using a spectrophotometer (Table 1).

**Table 1:** The mean value of color change (∆E) of the studied samples after their immersion in different solutions

Material	Color change (Mean ± SD)					
	Distilled water		Coca-Cola		Tea	
	Day 7	Day 30	Day 7	Day 30	Day 7	Day 30
Micro-hybrid	3.25 ± 3.37	3.33 ± 1.09	1.42 ± 1.08	7.64 ± 3.59	13.57 ± 2.63	19.94 ± 2.74
Nano-hybrid	3.6 ± 0.53	6.32 ± 4.41	5.83 ± 1.12	12.19 ± 2.39	8.95 ± 1.65	15.7 ± 1.55
Glass-ionomer	4.38 ± 4.6	6.87 ± 5.38	7.7 ± 3.82	4.91 ± 1.48	7.48 ± 1.72	11.99 ± 4.25

Our results showed a significant difference in the specimen's color change with the Coca-Cola and tea-milk solution (*p* = 0.004 and 0.001, respectively) after seven days and (*p* = 0.003 and *p* = 0.005, respectively) after 30 days. However, no significant difference was seen in the samples immersed in DM after seven days (*p* = 0.859) and 30 days (*p* = 0.366) (Tables 2 and 3).

**Table 2:** ANOVA test shows color change after 7 days’ immersion in different solutions

Material	Difference	Sum of squares	*df*	Mean square	*F*	*P*-value
Distilled water	Between groups	3.378	2	1.689	0.154	0.859
	Within groups	131.482	12	10.957		
	Total	134.859	14			
Coca-Cola	Between groups	104.093	2	52.047	9.183	0.004*
	Within groups	68.016	12	5.668		
	Total	172.109	14			
Tea	Between groups	100.838	2	50.419	11.973	0.001*
	Within groups	50.534	12	4.211		
	Total	151.372	14			

*Significant difference

**Table 3:** ANOVA test shows color change after 30 days’ immersion in different solutions

Material	Difference	Sum of squares	*df*	Mean square	*F*	*P*-value
Distilled water	Between groups	36.165	2	18.083	1.094	0.366
	Within groups	198.337	12	16.528		
	Total	234.502	14			
Coca-Cola	Between groups	135.311	2	67.655	9.761	0.003*
	Within groups	83.177	12	6.931		
	Total	218.488	14			
Tea	Between groups	158.007	2	79.004	8.465	0.005*
	Within groups	112.000	12	9.333		
	Total	270.008	14			

*Significant difference

Regarding sample ∆L (lightness), positive ∆L designates lighter samples, while negative ∆L designates darker samples after immersion. In this study, the result shows that the lightness (∆L value) of all specimens became negative (darken) in the tea-milk solution, and there was less change in DW and variable values in Coca-Cola (Tables 3, 4 and 5). The highest value was observed in micro-hybrid 1-month post-immersion in tea-milk solution (− 12.16 ± 1.74) (Table 4).

**Table 4:** Mean values of color change of different filling materials after exposure to tea

Time-line	Micro-hybrid			Nano-hybrid			Glass-ionomer		
	∆L	∆a	∆b	∆L	∆a	∆b	∆L	∆a	∆b
Day 7	− 7.28 ± 1.09	8.9 ± 2.68	6.28 ± 3.76	− 6.34 ± 1.02	− 0.76 ± 0.56	5.84 ± 2.82	− 6.84 ± 1.23	1.92 ± 1.92	− 0.18 ± 2.15
Day 30	− 12.16 ± 1.74	11.4 ± 3.82	9.94 ± 4.0	− 9.2 ± 1.2	5.0 ± 3.75	10.78 ± 3.55	− 10.96 ± 4.22	4.48 ± 1.65	− 0.5 ± 1.3

**Table 5:** Mean values of color change of different filling materials after exposure to distilled water

Time-line	Micro-hybrid			Nano-hybrid			Glass-ionomer		
	∆L	∆a	∆b	∆L	∆a	∆b	∆L	∆a	∆b
Day 7	− 5.0 ± 2.68	0.78 ± 0.44	− 0.8 ± 3.92	1.02 ± 0.3	0.92 ± 0.08	3.3 ± 0.55	0.54 ± 6.31	0.02 ± 0.69	− 0.12 ± 2.13
Day 30	1.24 ± 2.15	2.2 ± 0.47	0.88 ± 1.13	− 2.2 ± 6.44	2.18 ± 1.33	2.4 ± 2.65	0.54 ± 6.31	0.02 ± 0.69	− 0.12 ± 2.13

Moreover, negative ∆a specify turning to green color, while positive ∆a specify turning to red. Our samples showed positive ∆a value for all specimens (Tables 4, 5 and 6) except for nano-hybrid specimens after seven days in tea-milk solution (− 0.76 ± 0.56) (Table 3). The highest value has been recorded by micro-hybrid composite in tea-milk solution after 30 days (11.4 ± 3.82) (Table 4).

**Table 6:** Mean values of color changes of different filling materials after exposure to Coca-Cola

Time-line	Micro-hybrid			Nano-hybrid			Glass-ionomer		
	∆L	∆a	∆b	∆L	∆a	∆b	∆L	∆a	∆b
Day 7	− 0.08 ± 1.48	0.34 ± 0.64	− 0.68 ± 0.6	− 0.14 ± 0.33	0.66 ± 0.05	5.78 ± 1.14	− 6.86 ± 3.05	2.2 ± 0.85	2.38 ± 2.58
Day 30	1.92 ± 8.36	2.4 ± 1.07	0.44 ± 1.69	5.66 ± 8.02	1.3 ± 3.22	7.6 ± 1.52	− 0.12 ± 2.13	2.04 ± 0.9	1.3 ± 2.81

Finally, positive ∆b means a change to yellow color, and negative ∆b implies a change to blue color. Most of our samples had a positive ∆b value (Tables 4, 5 and 6), except for micro-hybrid in DW (− 0.8 ± 3.92) after seven days and glass ionomer after 7 and 30 days (− 0.12 ± 2.13) (Table 5) with micro-hybrid in Coca-Cola (− 0.68 ± 0.6) after seven days (Table 6). Collectively, duration of time, material type, and solution type have a statistically significant effect on ΔE.

## Discussion

Used dental restorative materials are vulnerable to various adverse effects in the oral cavity, such as the impact of acidity of different diets and drinks. Moreover, the high frequency of dental problems claims the essential outlook on the degradation capacity of restorative materials to protect their quality [6, 23].

We used 3 different solutions to check the color stability of 3 restorative materials that are widely used in dental practice nowadays and realized that DM presented no noticeable color changes in the materials. In comparison, the tea solution developed significant color changes in the composites. These findings are similar to those found in Saudi Arabia [24] and Turkey [25], which found that tea was the most staining solution, while DW did not alter the colors of the composites. These outcomes approve that water absorption by itself does not change the composite stainability to a substantial level [26].

Rather than using DW as an immersion medium, Tanthanuch et al. [6] in Singapore reported a clinically perceptible color alteration (ΔE > 3.3) for various composites immersed in tea, wine, and coffee. However, Falkensammer et al., 2013 demonstrated that red wine was a more potent discoloration agent than black tea (ΔE > 5.5) for nano-hybrid and micro-hybrid materials [27], and Majeti et al. [28] stated that diet coke was more potent discoloration medium than green tea, but less potent than coffee.

On the other hand, the color change in used materials increased substantially over time, especially for tea and Coca-Cola, which agreed with results found by other authors who indicated that the degree of change in the used restorative materials becomes greater with duration and frequency of interaction with the beverages, so a decrease in the amount and frequency of beverage intake was recommended [4].

Furthermore, we found that nano-hybrid presented higher color changes than micro-hybrid, which is in line with the outcomes of research in Saudi Arabia, which mentioned that nano-hybrid had a higher ΔE value and discoloration with less color stability despite having a higher degree of conversion compared to micro-hybrid, especially when immersed into coffee rather than tea or Coca-Cola [17]. However, Poggio et al. [29] in Italy excluded that there is no significant difference in discoloration ability between micro-hybrid and nano-hybrid composites in tea, and both increased staining. Silva et al. [30] showed that the Filtek Z350XT significantly stained higher than all other tested composites. Whereas Garcia et al. [31] reported that the color of restorative material changes with time, regardless of immersion solution (saliva, coke, or tea).

Generally, it has been stated that these outcomes can occur as nanomaterials have smaller particle sizes with a smoother surface that retains fewer surface stains [25]. Furthermore, it was suggested that any composite that can absorb water could also absorb other fluids, resulting in discoloration and reduction of its powered features due to polymer matrix degradation [32].

Another study demonstrated more discoloration of a nano-filled composite than a micro-hybrid which could be due to the composition of the resin matrix, as resin matrix also has a major effect on the color stability of composite resin. The conversion rate and chemical characteristics of the resin matrix modulate the discoloration. The composite resin contains TEGDMA in its composition. Therefore, it releases more monomers in the aqueous media than Bis-GMA- and UDMA-based composite resin, resulting in more remarkable color alteration [33].

It is difficult to explain which dental formulation is more crucial than others. But it can be solved after determining the weight criteria of different material performances and evaluating the ranking of the alternative dental composite [34, 35].

Regarding the study's limitations, we did not use saliva as an immersion medium due to infection control considerations. Additionally, the prepared discs did not look like a restoration from the geometrical perspective, but our results can be used to formulate designs for clinical experiments.

## Conclusions

Within the limitation of our study, we concluded that all materials used for this thesis showed undesirable color alteration after immersion in the tea-milk solution for one month. However, we realized that all used solutions (tea-milk solution, Coca-Cola, and distilled water) impacted on color stability of used restorative materials; but within a clinically acceptable range. Additionally, we found that micro-hybrid composite had high color stability compared to nano-hybrid and glass ionomer. Further study under the different conditions with other different esthetic restorative materials can be done in the future for better results.

## Data Availability

The datasets used and/or analyzed during the current study are available from the corresponding author upon reasonable request.
